# Neural Activity During Call Production in the Female Zebra Finch Homolog of the Male Forebrain Song System

**DOI:** 10.1111/ejn.70123

**Published:** 2025-04-30

**Authors:** Lisa Trost, Manfred Gahr, Andries ter Maat

**Affiliations:** ^1^ Department of Behavioural Neurobiology Max Planck Institute for Biological Intelligence Seewiesen Germany

## Abstract

Female zebra finches ( *Taeniopygia guttata* ) are unable to sing because of the vestigial development of forebrain song control areas such as the RA (nucleus robustus archistriatalis), a premotor nucleus of the song control pathway. In male zebra finches, RA is also involved in call‐based vocal communication in addition to song control. Here, we monitored the activity of RA neurons during vocal communication in freely behaving females using a miniaturized telemetric recording device combined with telemetric audio recording. Neurons in the RA region showed premotor activity associated with stack and tet calls, two innate short‐range social calls produced by both sexes. RA units were active when females called to respond to a male partner's call or to initiate a partner's call. However, spontaneous, regularly firing units, typical of male RA, were very rare in females or, when found, showed no association with vocal output. Despite the small number of adult female RA neurons, these neurons are not functionless, but are involved in call‐based communication.

## Graphical abstract

Although female zebra finches do not learn vocalizations, they possess brain structures analogous to those of males that use these structures to learn a large part of their vocal output, such as song. We wirelessly recorded neural signals from one of these brain areas, premotor nucleus RA, simultaneously with the vocal output from freely behaving females. These recordings show that females, like males, do use their “song system” during the exchange of stack and tet calls with their partners.

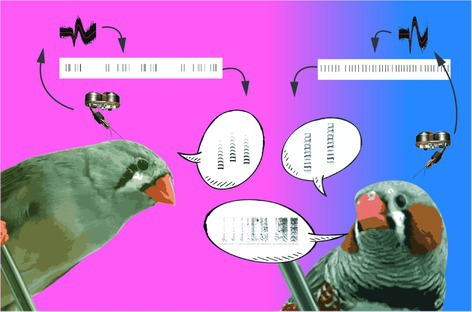

**Abbreviations**
dph: days post hatching
HVC: used as a proper name
ISI: interspike interval
PSTH: peristimulus time histogram
RA: nucleus robustus archistriatalis

## 1 Introduction

In songbirds, a network of brain nuclei, called the “song control system,” shows significant differences between the sexes. Even in species where both sexes sing and there is no morphological dimorphism, the song control nuclei differ considerably (Voigt and Gahr 2011). In a well‐investigated model species, the zebra finch ( *Taeniopygia guttata* ), large‐scale sex differences are found not only in the size and morphology of these nuclei, but also in their function. Male brains contain larger song nuclei than female brains and these nuclei are thought to be a prerequisite to learn and produce individual songs (Nottebohm and Arnold 1976; Nottebohm et al. 1976). Adult females have a less developed song control system with smaller nuclei and some axonal connections are missing (Wang et al. 2014, 2019). For example, premotor nuclei HVC and RA are five times larger in males than in females and the axonal connection between these nuclei seems to be less developed in the female brain (Konishi and Akutagawa 1985). Because of this, females are likely to produce only innate species‐specific calls (Güttinger and Nicolai 1973; Zann 1996), but no song (Nottebohm and Arnold 1976; Simpson and Vicario 1990).

Rudimentary brain areas are in general expected to be non‐functional, an evolutionary remnant. Nevertheless, despite the much smaller neuron populations, the overall connectivity of the song‐controlling areas of the forebrain of female zebra finches is similar to that of male zebra finches (Gurney 1981; Gahr 2007; Shaughnessy et al. 2019).

Male zebra finches use their song control nuclei not only for the production of songs but also for call‐based communication (Ter Maat et al. 2014). In males, RA and HVC neurons are involved in the timing of specific call types during call exchange (Ter Maat et al. 2014; Benichov et al. 2016; Ma et al. 2020) and HVC neurons additionally show an anticipatory function during call exchange (Benichov et al. 2016; Ma et al. 2020). Here, we aim to test a functional role for female zebra finch neurons homologous to those in male song control areas. Zebra finch calls are important for individual recognition (Vignal et al. 2004; D'Amelio et al. 2017a; Elie and Theunissen 2018) and pair formation (D'Amelio et al. 2017b), which leads to higher breeding success (Gill et al. 2015). As communication partners, females would need similar neural control mechanisms as males because communication requires recognition and detection of the partner's calls as well as exact timing and anticipation of possible answers in a certain context. Benichov et al. (2016) showed that chemical lesions of RA reduced response timing ability to played‐back calls in both male and female zebra finches and impeded their capacity to avoid jamming in call usage. These lesion experiments also showed that call production per se does not require RA because calling persisted (Simpson and Vicario 1990; Benichov et al. 2016).

Taking all this into account, we hypothesize that female zebra finches will use RA to control innate calls during vocal communication. Based on our former studies on males, we focused on the comparison of male and female RA neuron activity during call‐based communication.

We performed extracellular recordings from RA neurons in free‐moving female zebra finches using miniature amplifier transmitters to record the spiking activity. Synchronously, the vocal output of all male and female group members was captured by miniature microphone backpacks to compare the association of spiking activity and vocal output in females with that of males.

## 2 Materials and Methods

### 2.1 Animals

Experimental animals were adult female and male zebra finches bred in our Seewiesen colony and housed in single‐sex aviaries until the experiment. For the experiments, we kept birds in groups of four, two females and two males to offer a choice of partners. The groups were housed in aviaries of 1 m × 1 m × 1 m with a transparent perspex top to ensure signal transmission. The aviaries were equipped with four perches and one or two natural branches. Food and water were offered ad libitum, as well as sand bath, grit, and lime shells. We kept the birds in a 14/10 light/dark cycle (fluorescent lamps), at a room temperature of 22°C to 24°C and a humidity of 50%–70%.

In the experiment, all four members of a group carried wireless microphone transmitters to record the vocal output. We surgically implanted a tungsten electrode in the right hemispheric RA of one female in each group. The electrodes were connected to a wireless transmitting high‐impedance amplifier to record electrical activity in RA while free moving (Ter Maat et al. 2014). Experiments started 4 days after backpack mounting to ensure that all birds got used to carrying the extra weight (Gill et al. 2016). Permission to conduct the animal experiment, in accordance with the German Animal Welfare Act, was granted by the Government of Upper Bavaria under File Number 55.2‐1‐54‐2532‐148‐2015.

### 2.2 Implantation of Deep Electrodes

The birds were anesthetized using isoflurane inhalation (initial 1.5%, maintenance 0.8%–1% with a 400 mL/min O^2^ flow). The body temperature of the birds was kept stable using a warming pad (110 × 77 mm, 12 V, Thermo GmbH). For analgesia, we used metamizole (100–150 mg/kg, i.m.) 20 min before starting and meloxicam after finishing the surgery (1.5–2 h). The feathers over the surgical area were plucked, the skin disinfected and treated with liquid procaine (2%), and an incision was made over the surgical field. After carefully pushing the skin aside, a window was opened in the skull over the bifurcation of the midsagittal sinus, which served as reference. A second window was then made over the coordinates of RA. After opening the dura mater, a 2 MΩ tungsten electrode (FHC, Bowdoin, USA) was then lowered into RA using a Luigs and Neumann SM‐5 manipulator. The coordinates were based on the coordinates of the male RA. The reference electrode, a platinum wire (0.025 μm, Goodfellow), was fixed between dura mater and skull. The connectors of reference and recording electrodes were fixed in place using dental cement (Tetric evoflow refill, A1, Ivoclar Vivadent). The connectors serve as a support for the transmitter. During insertion of the electrode, electrical activity was amplified by a DAM 80 (AC Differential Amplifier, WPI) and monitored online using a continuous update of the ISI plot of Schmitt‐triggered spikes. In a previous study in males (Ter Maat et al. 2014), we identified RA unit activity by the typical ISI histograms of the spikes (Hahnloser et al. 2006). This spike pattern was rarely found in females.

The electrode positions were verified by electrical lesions at the end of the experiments. Subsequently, brains were sectioned and Nissl‐stained.

### 2.3 Neuronal Recording and Analysis

To record the electrical activity of RA in free‐moving female zebra finches, we used a lightweight (1.0 g) device that contains an amplifier (10–10,000 Hz) and a transmitter. The transmitters used in the current study are a further development of the device we used earlier with zebra finch males (Schregardus et al. 2006; Ter Maat et al. 2014). Their transmitted power was increased to yield a range of > 10 m, with a battery life of over 7 days. Tuned crossed‐yagi antennae (Winkler Spezialantennen, Kreuzdipol 300, directional antenna for 300 MHz, clockwise) received the wireless signals. The signal from the antenna was amplified by 18 dB (TVS 14‐00 axing, Goobay, Germany), split (BE 2‐01 premium‐line, Switzerland) and fed into up to eight AOR 8600 receivers (AOR, Ltd., Japan) to permit receiving the signals of eight transmitters. The receivers were modified to handle 12 kHz audio bandwidth. For the digitization of the signals, an eight‐channel audio A/D converter was used (M‐Track Eight, M‐Audio, USA; sampling rate: 22,050 Hz). All digitized signals were synchronously recorded as 4‐h wave files by a multichannel software (16 bit, 22,050 Hz, ASIO, Germany; Hoffmann et al. 2019).

To reconstruct the pattern of electrical spiking activity from probably multiple units in a given recording, each event in the recorded wave file above threshold was captured by peak detection and written to a record consisting of a timestamp (float) followed by 64 integers. Waveforms were then sorted using a k‐means sorting algorithm and further analyzed using custom software written for analysis of long, extracellular spike recordings with changing signal‐to‐noise ratio, as reported earlier (Jansen and Termaat 1992; Jansen et al. 2005).

### 2.4 Vocal Recordings and Analysis

To record the vocal output, we used in‐house developed wireless microphone backpacks (microphones: Knowles Electronics, FG23329). The microphone was attached to the circuit‐board and covered with shrink tubing. Another layer of silicon tubing with small openings allowed us to attach a string in two loops. These loops were placed around the legs and kept the transmitter on the back of the bird, with the microphone facing the body to enhance the specificity of the recording. After 4 days of accustoming to the backpack, the birds moved and vocalized like normal again (Gill et al. 2016) and recording was started. The recordings consisted of 4 h sessions digitized at 22,050 Hz. Vocal signals were fed into the same system as the neuronal signals and recorded synchronously.

The repertoire of both sexes consists of several call types, like stack, tet, hat, kackle, and distance calls (Elie and Theunissen 2016; D'Amelio et al. 2017b). Not all females in our study used all call types during the experiment and typically communicated with stack or tet or both calls. Therefore, we focused our analysis on these two call categories. Stack calls are harmonics with no or very low frequency modulation lasting up to 100 ms, while tet calls are shorter in duration with deeper frequency modulation (Ter Maat et al. 2014). We recorded the vocal output of each group continuously for up to 7 days before and at least 3 days after the surgery of each experimental female. Recordings before the surgical procedure were used to determine a possible communication between females and males and assess calling rates. These were not different between sexes for both tets and stacks (Figure S1A).

The vocal output of target females and corresponding males of each group was sorted into distinct call types using Sound Explorer (written by R.F. Jansen; Ter Maat et al. 2014). The temporal association between the vocalizations of each experimental female and the respective male of a group was determined using cross‐correlations between the peristimulus time histograms (PSTHs) (Abeles 1982). Following the definition from our work on male zebra finches, a rise in the call count exceeding confidence intervals in half a second before or after the partner's call is considered as communication between the two birds (Ter Maat et al. 2014). We examined all combinations of calls in the communication of females and males (stack–stack, tet–tet, stack–tet, and tet–stack).

### 2.5 Association Between Neuronal and Vocal Signals

The data sets of the experimental animals were each obtained from a single 4‐h daytime recording. To show the association between neuronal activity and vocalization of each experimental female, we aligned clustered unit activity to the onset of sorted innate short, soft calls (stack and tet calls) using PSTHs. To determine the activity in RA during and after call production as a function of whether the call was an answer, was answered or had no association with the male call, as well as the type of call (tet or stack), we fitted a model that included animal‐ID as a random factor. We first constructed a PSTH (400 bins of 10 ms, 200 before and 200 after call onset) for the levels of each factor, i.e., female call type (stack or tet) and type of interaction (answer, answered, none). From each PSTH, the probability density function was used to calculate the average density of the bins 199–203 (10 ms before to 40 ms after call onset) and bins 225–250 (25–50 ms after call onset), representing early and late activity. The statistical test was done in JMP12. Testing was performed separately for early and late responses. The model was, in R‐notation: response ~ (female call type) * (interaction type) + 1/animal ID.

## 3 Results

### 3.1 Electrode Placement

**FIGURE 1:**
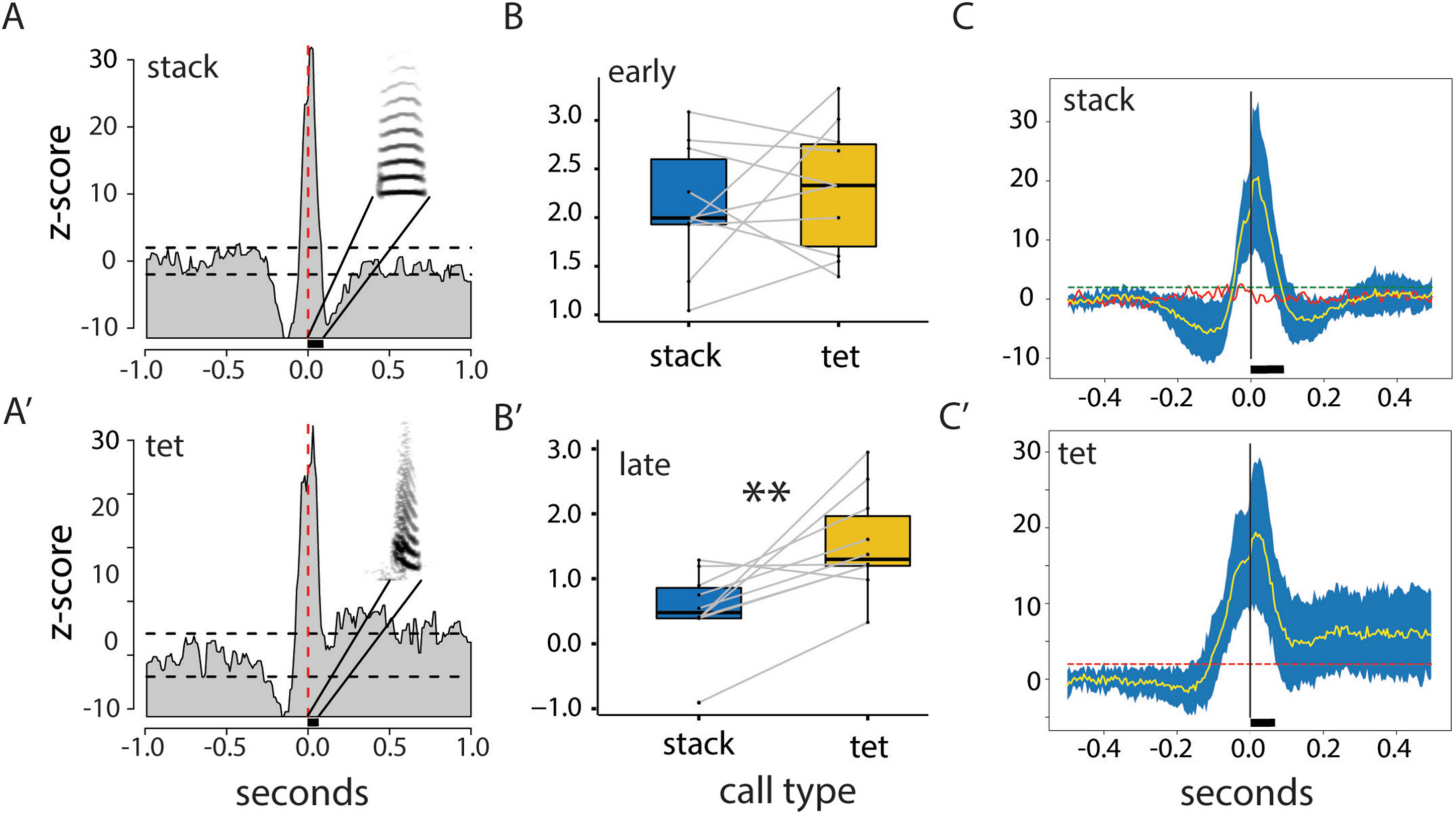
Neural activity is associated to self‐produced calls in females. (A, A′) PSTHs of multiunit neural activity aligned to the onset of stack calls (A) and tet calls (A′). Both neural and vocal signals were synchronously recorded from Female 7. Stack and tet calls of this female are shown in the plots. The lengths of the black bars below the plots show their durations. (B, B′) Comparison of spiking activity following stack (B) and tet calls (B′) in 11 female zebra finches (10 ms/bin). (B) Averages of z‐scores were calculated from call onset to 125 ms after onset (10 ms/bin; early). (B′) Averages of z‐scores were calculated from 150 to 750 ms after the onset (10 ms/bin; late) of either call type. Spiking is clearly more intense following tet calls than following stacks (*p* < 0.005; animal ID as random factor). (C, C′) Similarity of female neuronal firing patterns during call production. Mean (yellow line) and standard deviation (blue) of the PSTHs in 11 females during stack (C) and tet (C′) calls. (C) The PSTH of Female 23 (red line) has no clear association with her stack calls and is not included in the calculation of the standard deviation. (C′) Female 23 did not produce any tet calls. The black bars show the average durations of stack and tet calls.

Electrodes were placed by majority in RA in strict sense (RA‐s.s.) or in the outer rim of RA (RA‐area within 50–100 μm). To verify the recording of RA units, we compared the spiking pattern between electrode placements in the RA‐s.s. and in the RA‐area (Figure S2A) and found no difference in the associated neural signal for both call types (Figure S2B). One female in which the electrode was placed more than 500 μm off the medio‐lateral axis did not provide a neural association to her own vocal output and was excluded from further analysis (Figure 1C; Female 23).

### 3.2 General Vocal Interaction

**FIGURE 2:**
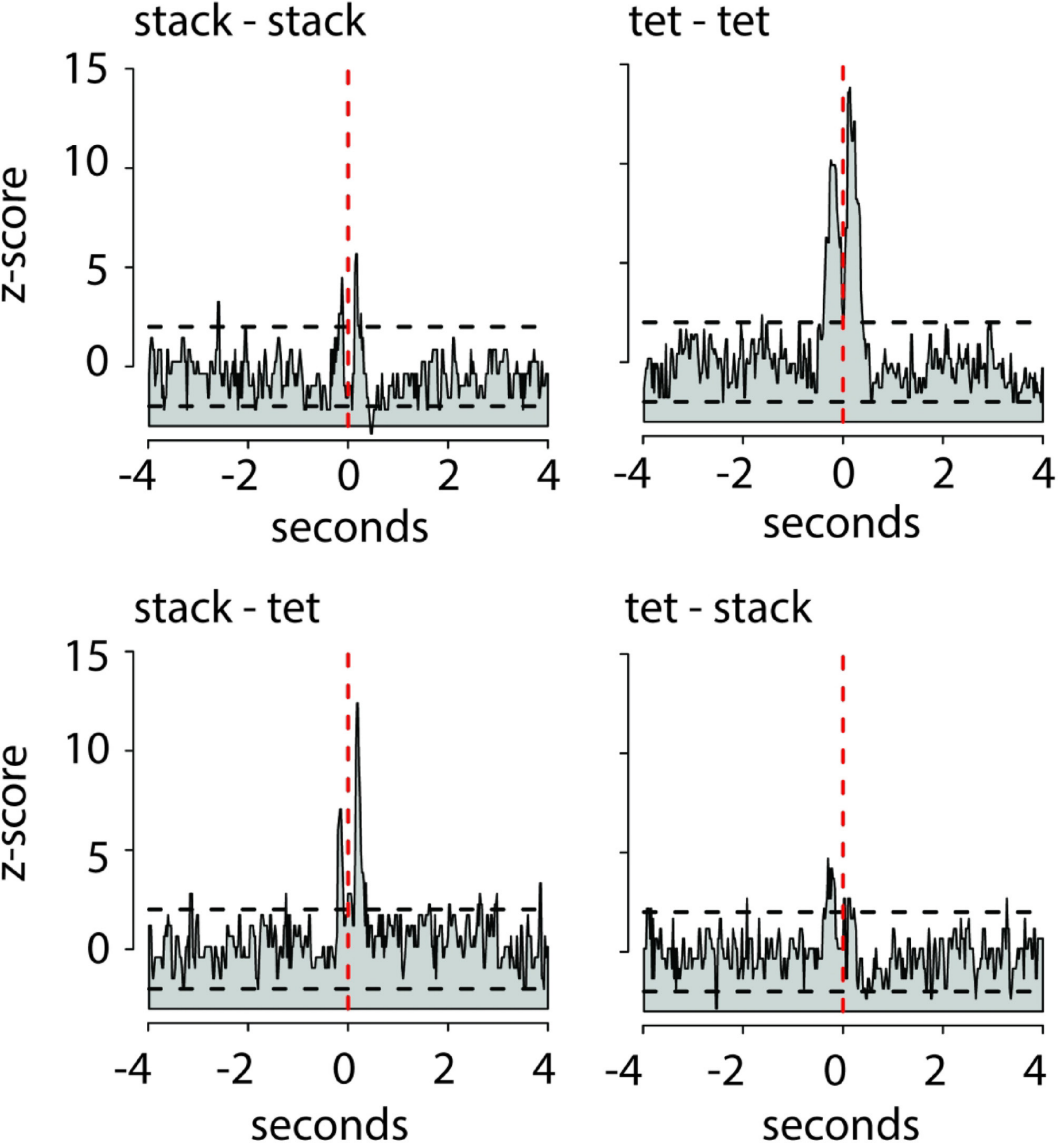
Vocal communication patterns between males and females. All possible combinations of stack and tet call communication between Female 4 and Male 4 in a 4‐h recording period, shown as peristimulus‐time‐histograms (PSTHs), where the onset times of the male calls are aligned to the onset times of the female calls, which are aligned on zero, represented by the red dashed line. The title of each panel gives the call combination with female call type first and male call type second. Z‐scores of male stack calls were calculated from 4 s before and after onset of female stack calls. The horizontal dashed lines represent the z‐scores of ±2. The probability of male calls occurring within half a second before or after a female call is clearly and significantly increased.

In our group setup, experimental females produced 1029 ± 721.6 stack and 1105 ± 887.7 tet calls in a 4‐h period. Similarly, experimental males produced 1365 ± 992.3 stack and 1089 ± 1054.4 tet calls in the same time span (Figure S1A). Female and male calling rates did not differ significantly (log‐transformed rate; REML with animal ID as random factor; call type: *p* = 0.441, gender: *p* = 0.448, interaction: *p* = 0.823). Two females (# 1, 2) did not show antiphonal calling with any of the group members, whereas all other females started vocal interaction with one of the males in the group. The pairs rarely used all possible call combinations to communicate (Figures 2 and S4).

### 3.3 Calling Related Firing Patterns in Female RA

**FIGURE 3:**
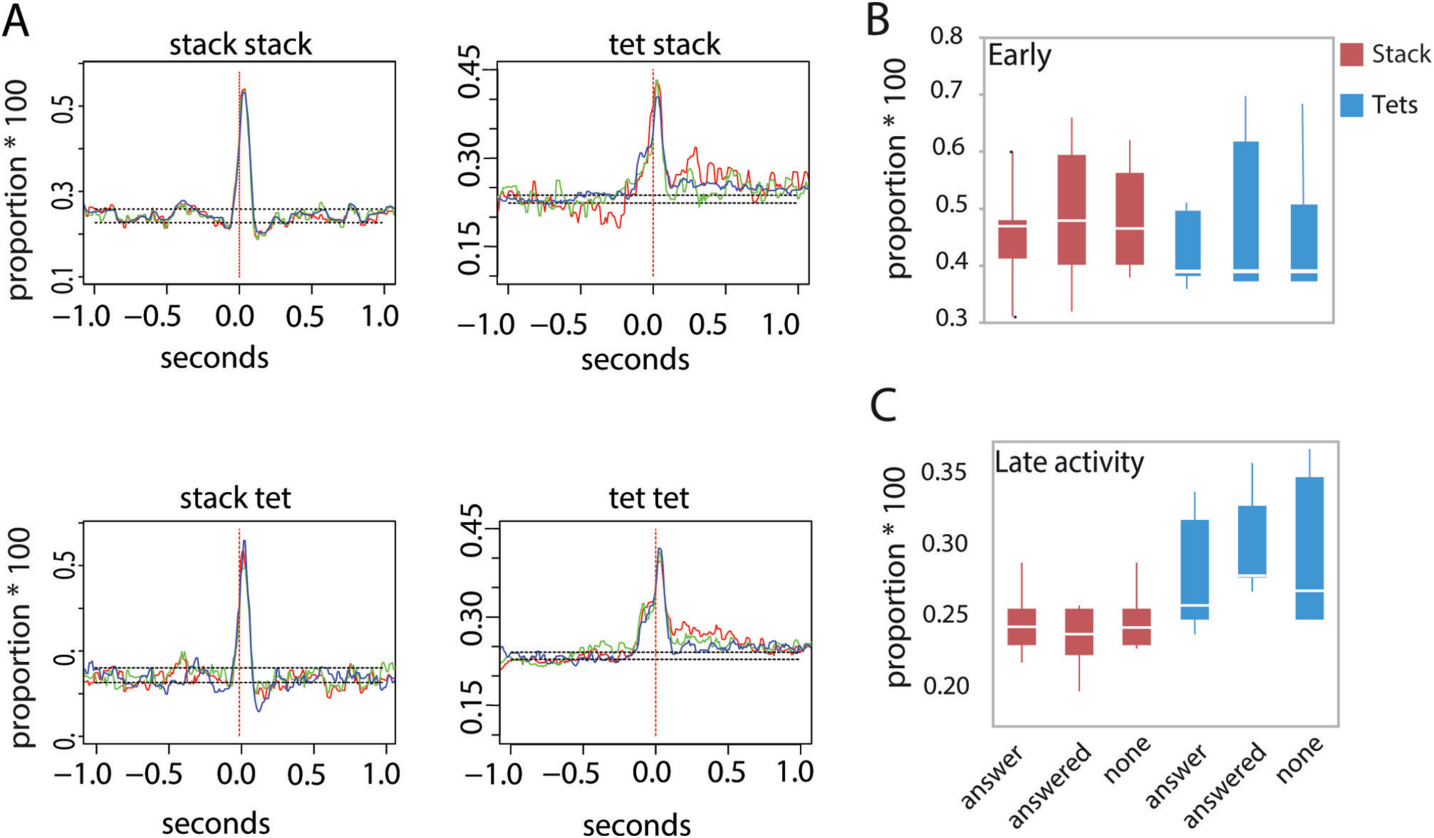
Neuronal activity during stack or tet calls used in different contexts. Relationship between neuronal activity and calling context. Stack and tet calls in four different female–male communication combinations (stack–stack, stack–tet, tet–tet, and tet–stack), are sorted in three context‐groups: Female calls “answered” (red lines) by a male call, female calls used as an “answer” (green lines) and female calls used in “none” (blue lines) of these contexts. Neuronal signals underlying the respective call category are aligned to the onset of all calls in a peristimulus time histogram. (A) Results for one bird, Female 4. The superimposed curves show no clear difference between contexts. (B) Early neuronal activity associated with stack and tet calls. The mean proportion of spikes (times 100) in a period from 10 ms before to 40 ms after call onset. With stacks, the early activity was higher than with tets (0.458 ± 0.091 vs. 0.443 ± 0.106; *p* < 0.0001). There was no significant effect of context (*p* = 0.356) or interaction between call type and context (*p* = 0.885). (C) Late neuronal activity associated with stack and tet calls. Here, the late activity (250–500 ms after call onset) was lower with stacks than with tets (0.245 ± 0.020 vs. 0.287 ± 0.040; *p* < 0.0001). Again, there was no significant effect of context (*p* = 0.696) or interaction between the two factors (*p* = 0.102).

Neuronal activity showed a clear motor pattern, with a pronounced change in neural activity before, during, and after stack and tet calls of the focal female (Figures 1A,B and S3). In all 11 females, we found a clear association between irregular units and the onset of the respective female stack and tet calls with a change in spiking activity (z‐scores > 1.73) within 0.5 s before and after the onset of either stack or tet call (Figures 1A–C and S3). Interestingly, increased firing rates took longer to return to baseline after tet calls than after stack calls (*p* < 0.005) (Figure 1A′,B′,C′). In eight females (# 1, 4, 5, 6, 7, 11, 14, 20), the analyzed multiunits associated with stacks showed an inhibition before call onset and excitation during the stack call. In the remaining three females, the neural pattern showed no pre‐inhibition, but only excitation during the stack call. We did not find a consistent explanation for the differences in inhibition patterns between the females. For the tet calls, pre‐inhibition occurred in five females (# 2, 7, 11, 14, 20) followed by excitation during the call, whereas in the remaining six females (# 1, 4, 5, 6, 8, 15) the neural signal showed solely excitation during the calls (Figure S3). However, the type of female call was significantly different in the late neuronal activity. Early activity with stacks was higher than with tets (stacks: 0.458 ± 0.091; tets: 0.443 ± 0.106; *p* < 0.0001). Late activity with stacks was lower with stacks than with tets (stacks: 0.245 ± 0.020; tets: 0.287 ± 0.040; *p* < 0.0001; Figures 1B,B′ and 3B,C).

### 3.4 Absence of Contextual Dependency

In most cases, the communication patterns showed asymmetry; for instance, one partner answered more often than received an answer, which was reflected in one‐sided call distributions in the histograms. Because not every call of partners was answered by the partner, we assigned calls to one of the categories: “answer”, “answered”, and “none” Answers needed to occur within 0.5 s of a call. The association with the neural signal showed that regardless of which category (answer, answered, none) of stack or tet calls was used, the maximum proportion of RA spiking activity did not significantly change (*p* = 0.528, JMP: response‐log transformed = combination * category + animal ID; Figures 3A and S5). Both with early and late neural responses, the factor “combination” (answer, answered, none) was never significant (Figure 3B,C).

### 3.5 Regular Firing RA Neurons Are Very Rare in Females

**FIGURE 4:**
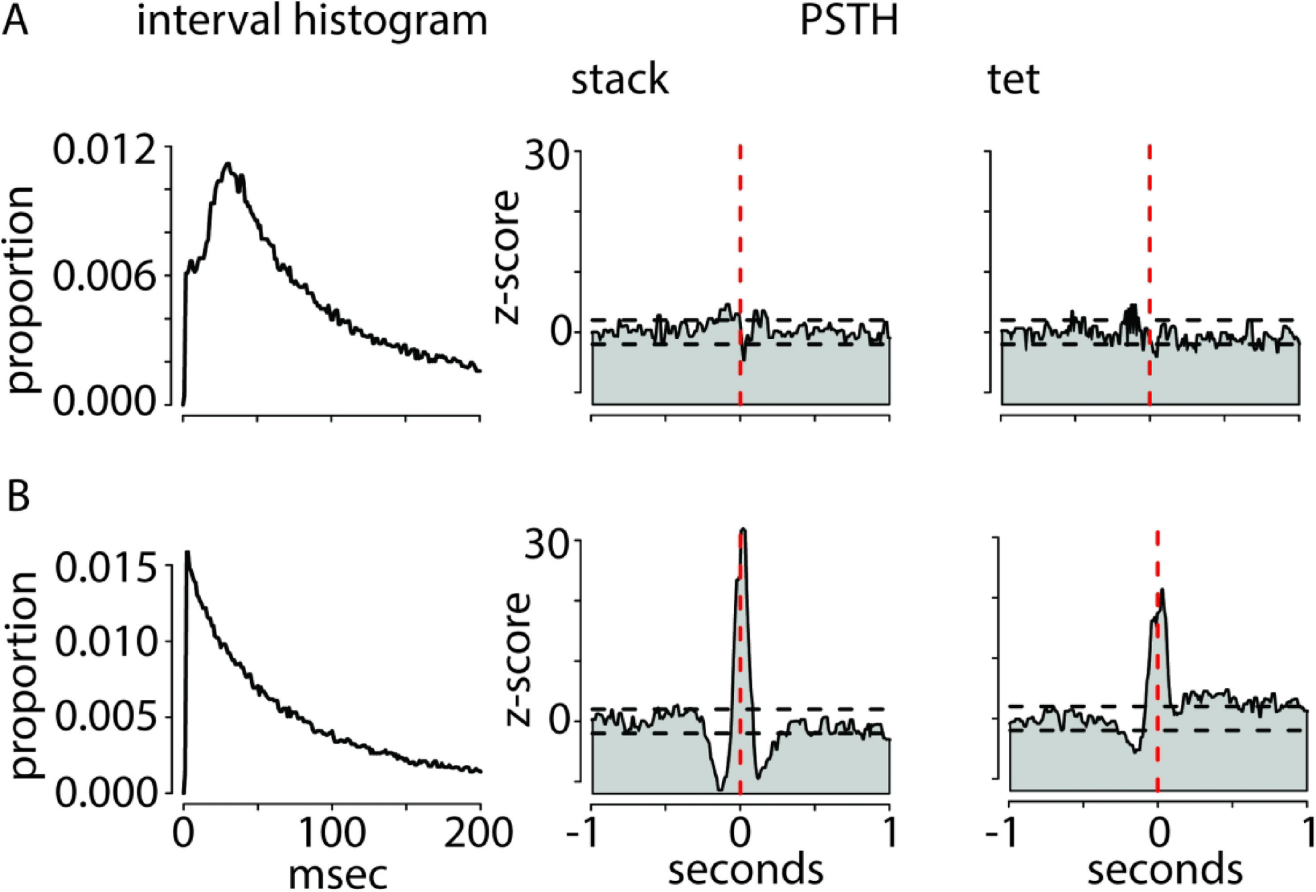
Regular and irregular firing RA neurons. Interspike interval of (A) a regular firing neuron and (B) irregular units (multiunit) both sorted out of the neuronal signal of Female 7. (A) Peristimulus time histograms of stack and tet show a lack of association between neuronal signal and onset of either call type. (B) Stack and tet peristimulus time histograms represent a clear link between neuronal signal and the onset of both call types.

In a previous study on male zebra finches, we identified RA units during surgery by their spontaneous, regular firing (Ter Maat et al. 2014). Further, spontaneous regular firing is a characteristic feature of RA projection neurons that transmit song information to the vocal and respiratory organs in behaving male zebra finches (Spiro et al. 1999; Hou et al. 2012). These patterns were also looked for in the experimental females. Only in three (# 7, 14, 15) of the 11 females, regular units were extracted from the neural recordings. In only one of the females (# 7), a regular firing unit was recorded under anesthesia and in the free‐behaving condition (Figure 4A,B), the other units occurred only under anesthetized conditions during surgery (# 14, 15). None of these regular units showed a clear association with the vocalization of the respective female, be it playback of calls or self‐produced calls (Figure 4A). This result is contrary to the abundance of regular firing neurons in male RA. There, they are usually associated with song (Hou et al. 2012) and call production (Ter Maat et al. 2014).

## 4 Discussion

### 4.1 RA Activity Is Associated With Calls in Females

Our recordings from premotor nucleus RA of female zebra finches reveal a clear association between neuronal activity and females' vocal communication with innate calls, the tet and the stack call. The firing patterns in RA differed between the call types. With stacks, firing decreases first, before the burst that is associated with call onset and duration and returns to resting level after a downward excursion. On the other hand, the tet call is associated with a delayed return to resting level. Earlier, we defined the difference between tets and stacks of male zebra finches based on their duration and the degree of frequency modulation (Ter Maat et al. 2014; D'Amelio et al. 2017b). The current result strengthens the classification of these calls.

Recent studies have shown that female zebra finches engageed in call communication with a playback are able to flexibly adjust their response timing to avoid jamming (Benichov et al. 2016). During pair bonding, both males and females gradually increase the symmetry between answered calls (D'Amelio et al. 2017b), indicating a learning process. Lesion studies show that the hindbrain vocal areas are sufficient for the production of the temporal‐spectral features of the calls of zebra finches (Simpson and Vicario 1990; Benichov and Vallentin 2020). However, precise timing of calls during vocal exchanges needs an intact forebrain. Our data, together with the impaired call timing of RA‐lesioned females (Benichov et al. 2016), suggest that female RA neurons are relevant for the call timing during vocal exchanges. The arcopallial area surrounding RA obtains input from Field L, a primary auditory cortex equivalent of birds, in the male zebra finch (Vates et al. 1996; Shaevitz and Theunissen 2007). Such auditory input to the arcopallium might be relevant for the RA to induce vocal responses. Alternatively, the cognitive processes to time the calling in females may take place in the HVC circuit, since Benichov and colleagues have shown in male zebra finches that blocking HVC interneurons leads to changes in the timing of the vocal response (Benichov and Vallentin 2020).

The association patterns between multiunits and females' own calls are very uniform in all of our experimental females, whereas male zebra finches revealed a number of different correlation patterns of single, regular firing units and stack calls, like multi‐phasic, bi‐, or monophasic excitation associated with their own stack calls (Ter Maat et al. 2014). These differences between males and females might be reflected by differences in the properties of RA circuits and neurons.

### 4.2 Sex‐Specific Properties of RA Neurons

In the rare cases where we found regular firing neurons in female RA, they were never associated with the vocal output of the respective female. This result was striking because regular units in males were, almost without exception, closely linked to male song or call production (Ter Maat et al. 2014) and are thought to be the motor basis of vocal output (Nottebohm et al. 1976). In male zebra finches, the ratio of regular to irregular firing RA neurons is 30:1 (Spiro et al. 1999). This is clearly not true for females. In spite of using the same spike‐sorting method as in the male study (Ter Maat et al. 2014), we could not separate regular firing neurons from the multiunit signal in females. In male zebra finches, the feature of regular firing develops in RA from Day 30 to 40 post‐hatching, when female RA cytoarchitecture tends to regress remarkably (Nixdorf‐Bergweiler 1996). Nevertheless, regular firing neurons occur in juvenile females (20–50 days post‐hatching) (Adret and Margoliash 2002). In addition, R.F. Jansen and M. v. d. Roest found such regular firing neurons in RA slices of ~50 dph females (personal communication). Apparently, regular firing features are lost during later ontogeny. Because we found a clear association between multiunit activity and calling in all of our experimental females, we propose that this activity represents the motor basis for timing and appropriate usage of different call types in female zebra finches. Hence, we assume that the regular firing units have consequently lost their projecting ability or never possessed it in female zebra finches.

Male and female RA's differ in several ways. (1) Male zebra finches receive more excitatory synaptic input to their projecting neurons than females (Wang et al. 2014). (2) Stimulation of the male HVC elicits a biphasic response mediated by glutamate in RA but results in only a slow, single‐phase response in the female RA (Wang et al. 1999). (3) Blocking GABAergic inhibition enhances RA activity in females but not in males (Wang et al. 1999). (4) Ultrafast spiking bursts driven by sodium resurgent currents are predominantly found in zebra finch males (Zemel et al. 2021). To this list, we add the lack of regular firing RA neurons in females. Female RA activity is clearly contributing to the timing of calling but is not sufficient for singing. Therefore, it is tempting to speculate that the lack of the regular firing neurons explains the lack of song development.

### 4.3 Female Specific Development of RA

Sex differences in cellular and cytoarchitectural properties of the song control system in songbirds are among the largest in the animal kingdom (MacDougall‐Shackleton and Ball 1999; Gahr 2007). However, even in species where females and the males produce songs of equal quantity and complexity, sex differences in the song control system are always male‐biased (Gahr et al. 1998; Lobato et al. 2015; Ko et al. 2021). Species in which adult females do not sing and cannot be induced to sing provide the clearest example of the complex differences in the vocal control system between the sexes. Examples are the zebra finch (Nottebohm and Arnold 1976), the Bengalese finch (Tobari et al. 2005) and the white‐throated sparrow (Devoogd et al. 1995). However, here we demonstrate functional, vocal‐related neurons in a presumed rudimentary song control region, the RA of female zebra finches. This suggests that sexually dimorphic regressed brain areas are rather sex‐specific adaptations to the vocal communication system of certain species than evolutionary remnants. Assuming that the control of call timing is a function of male and female forebrain song control areas of songbirds in general, these brain areas control more than just song.

## Author Contributions

**Lisa Trost:** conceptualization, data curation, formal analysis, investigation, methodology, project administration, supervision, writing – original draft, writing – review and editing. **Manfred Gahr:** conceptualization, resources, writing – review and editing. **Andries ter Maat:** conceptualization, data curation, formal analysis, investigation, methodology, project administration, supervision, writing – original draft, writing – review and editing.

## Conflicts of Interest

The authors declare no conflicts of interest.

### Peer Review

The peer review history for this article is available at https://www.webofscience.com/api/gateway/wos/peer‐review/10.1111/ejn.70123.

## Supporting information


**Figure S1.** (A) Log‐transformed count of all stack and tet calls averaged over 4 h across all females and males. Call production was not different between both. (B) Relationship between stack call duration and period of elevated spiking of 11 females.
**Figure S2.** Lesions and respective firing pattern. (A) Two sagittal sections (25 μm, Nissl‐staining) showing RA and the lesion from Female 2 and Female 4. Visible are the electrical lesions as an estimate of the electrode placements. The two examples show placements in RA‐s.s. (Female 2) and in the RA‐area (Female 4). The yellow circles mark the visible borders of RA. (B) Spiking activity aligned to stack and tet onset is very similar between electrode placements inside RA‐s.s. and in the area outside but close to RA (RA‐area). With stacks, spiking rate decreases then increases steeply before call onset and settles back to pre‐call levels after the call. This occurs in both situations. With tets, the firing rate before and during the call follows a trajectory like during stack calls, but following the call it remains higher than baseline, both with the electrode inside or near RA.
**Figure S3.** Spiking activity aligned to stack and tet calls of all females. Neuronal multiunit alignment to the onset of stack and tet call of each experimental female presented in the form of PSTHs, with 1 s (x‐axis; 10 ms/bin) before and after the onset of either call type (red dashed line on zero). The y‐axis shows z‐scores of the multiunit activity. The horizontal dashed lines represent the ± 2 × z‐score. For Female 23 not shown.
**Figure S4.** Antiphonal calling analysis of all pairs. All possible combinations of stack and tet call communication between each experimental female and the respective partner are shown as PSTHs, where the onset times of the male calls are aligned to the onset times of the female calls (red dashed lines). The female call type is given as first, the male call type as second in the title of each panel. Z‐scores of male stack calls were calculated from 4 s before and after onset of female stack calls. The horizontal dashed lines represent ± 2 × z‐score. Combinations that were rarely used by the birds are shown in opaque line thickness.
**Figure S5.** Neuronal activity during stack or tet calls used in different contexts. Calls (stacks and tets) of all females, that established antiphonal communication with one of the males, are divided in three groups: Female calls “answered” by a male call, female calls used as an “answer” (green lines) and female calls used in “none” (blue lines) of these contexts. Neuronal signals underlying the respective call category are aligned to all three versions of usage in a peristimulus time histogram. Red lines: Neural 8 association to “answered calls,” green lines: neural association to “answer calls” and blue lines: neural association to calls in no recognizable context. The association between neuronal signals and, respectively, used call category are not different. Female 4 showed all possible combinations (stack–stack, stack–tet, tet–tet, and tet–stack), but at least one combination is presented for the other experimental females.

## Data Availability

The data utilized in this study are available in the open access research data repository Edmond of the Max‐Planck Society under https://doi.org/10.17617/3.1MXFYH.
