# Supplementary material for: Neural Activity During Call Production in the Female Zebra Finch Homolog of the Male Forebrain Song System
Source: Eur J Neurosci. 2025 Apr 30;61(9):e70123. doi: 10.1111/ejn.70123 (PMC12042645; doi:10.1111/ejn.70123)
Supplement: Supplementary file 1 — Figure S1. (A) Log‐transformed count of all stack and tet calls averaged over 4 h across all females and males. Call production was not different between both. (B) Relationship between stack call duration and period of elevated spiking of 11 females. Figure S2. Lesions and respective firing pattern. (A) Two sagittal sections (25 μm, Nissl‐staining) showing RA and the lesion from Female 2 and Female 4. Visible are the electrical lesions as an estimate of the electrode placements. The two examples show placements in RA‐s.s. (Female 2) and in the RA‐area (Female 4). The yellow circles mark the visible borders of RA. (B) Spiking activity aligned to stack and tet onset is very similar between electrode placements inside RA‐s.s. and in the area outside but close to RA (RA‐area). With stacks, spiking rate decreases then increases steeply before call onset and settles back to pre‐call levels after the call. This occurs in both situations. With tets, the firing rate before and during the call follows a trajectory like during stack calls, but following the call it remains higher than baseline, both with the electrode inside or near RA. Figure S3. Spiking activity aligned to stack and tet calls of all females. Neuronal multiunit alignment to the onset of stack and tet call of each experimental female presented in the form of PSTHs, with 1 s (x‐axis; 10 ms/bin) before and after the onset of either call type (red dashed line on zero). The y‐axis shows z‐scores of the multiunit activity. The horizontal dashed lines represent the ± 2 × z‐score. For Female 23 not shown. Figure S4. Antiphonal calling analysis of all pairs. All possible combinations of stack and tet call communication between each experimental female and the respective partner are shown as PSTHs, where the onset times of the male calls are aligned to the onset times of the female calls (red dashed lines). The female call type is given as first, the male call type as second in the title of each panel. Z‐scores of male [file EJN-61-0-s001.pdf]

Supplementary Figures

Figure S1

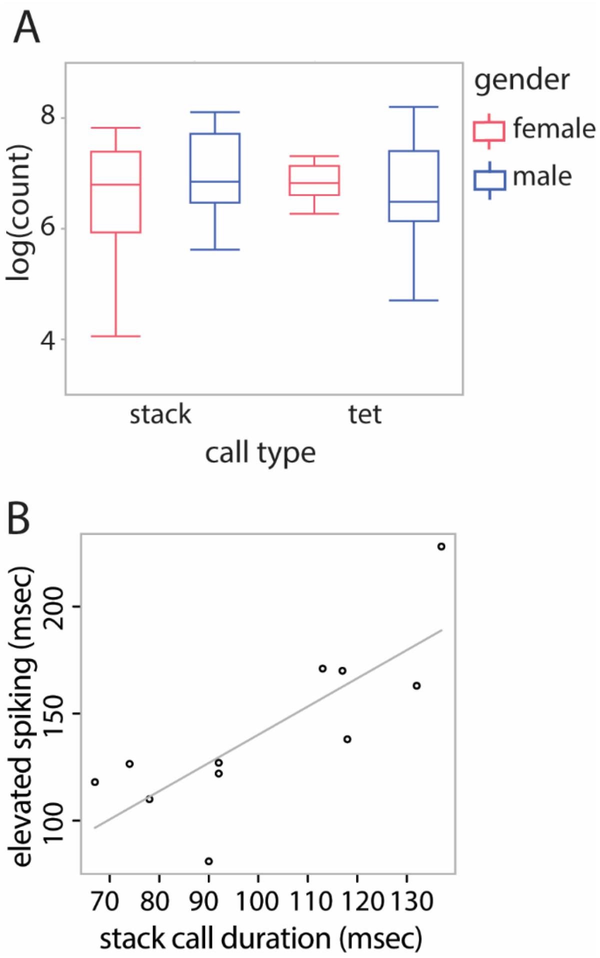

Fig. S1 A. Log-transformed count of all stack and tet calls averaged over 4 hours across all females and males. Call production was not different between both. **B.** Relationship between stack call duration and period of elevated spiking of 11 females.

Figure S2

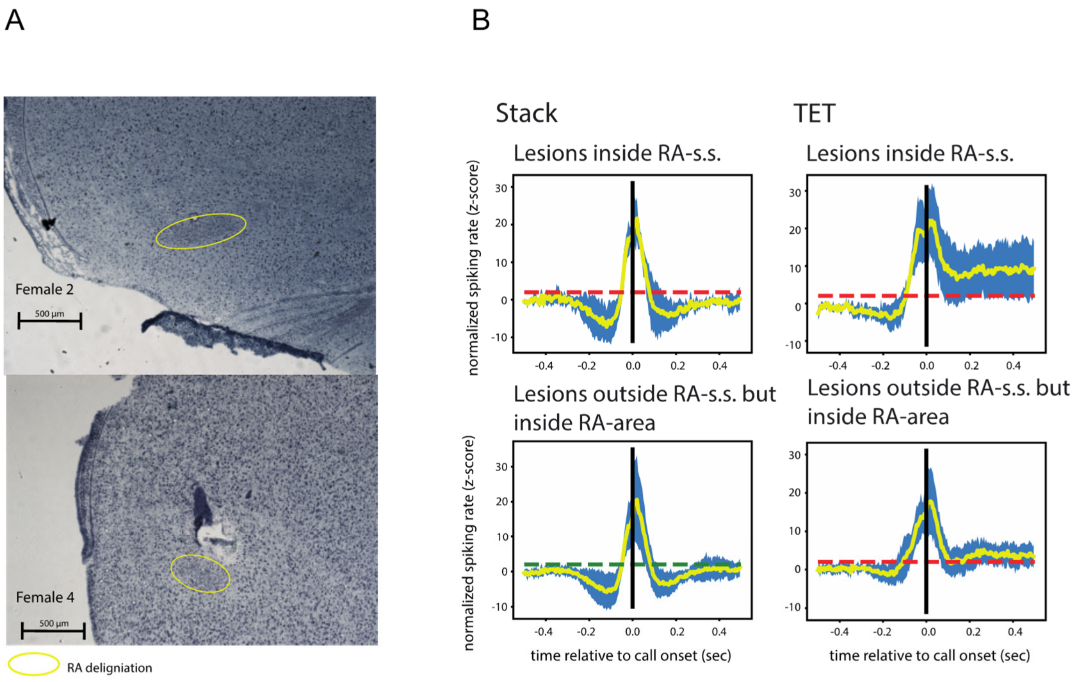

Fig. S2: Lesions and respective firing pattern. A. Two sagittal sections (25 µm, Nissl-staining) showing RA and the lesion from female 2 and female 4. Visible are the electrical lesions as an estimate of the electrode placements. The two examples show placements in RA s.s. (Female 2) and in the RA-area (Female 4). The yellow circles mark the visible borders of RA. **B**. Spiking activity aligned to stack and tet onset is very similar between electrode placements inside RA-s.s. and in the area outside but close to RA (RA-area). With stacks, spiking rate decreases then increases steeply before call onset and settles back to pre-call levels after the call. This occurs in both situations. With tets, the firing rate before and during the call follows a trajectory like during stack calls, but following the call it remains higher than baseline, both with the electrode inside or near RA.

Figure S3

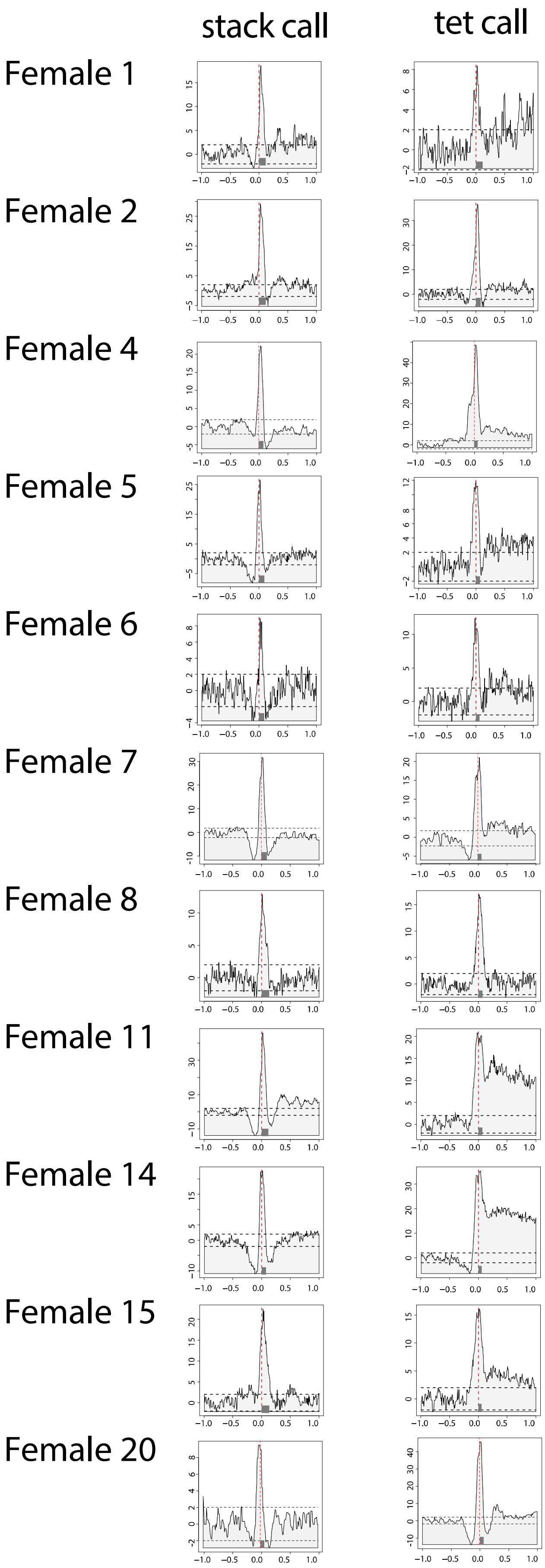
stack call
tet call
Female 1
Female 2
Female 4
Female 5
Female 6
Female 7
Female 8
Female 11
Female 14
Female 15
Female 20

Fig. S3 Spiking activity aligned to stack and tet calls of all females. Neuronal multiunit alignment to the onset of stack and tet call of each experimental female presented in the form of PSTHs, with 1 second (x-axis; 10 ms/bin) before and after the onset of either call type (red dashed line on zero). The y-axis shows z-scores of the multiunit activity. The horizontal dashed lines represent the ± 2 x z-score. For female 23 not shown.

Figure S4

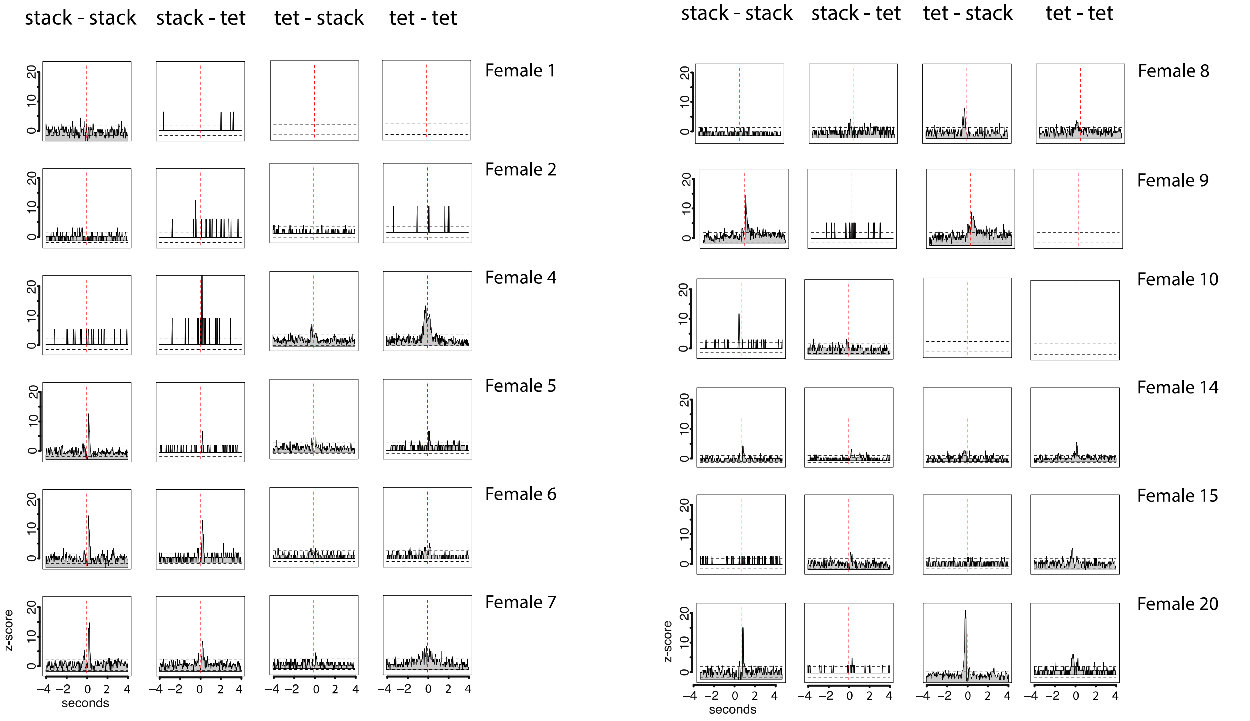

Fig. S4. Antiphonal calling analysis of all pairs. All possible combinations of stack and tet call communication between each experimental female and the respective partner are shown as PSTHs, where the onset times of the male calls are aligned to the onset times of the female calls (red dashed lines). The female call type is given as first, the male call type as second in the title of each panel. Z-scores of male stack calls were calculated from 4 seconds before and after onset of female stack calls. The horizontal dashed lines represent ± 2 x z-score. Combinations that were rarely used by the birds are shown in opaque line thickness.

Figure S5

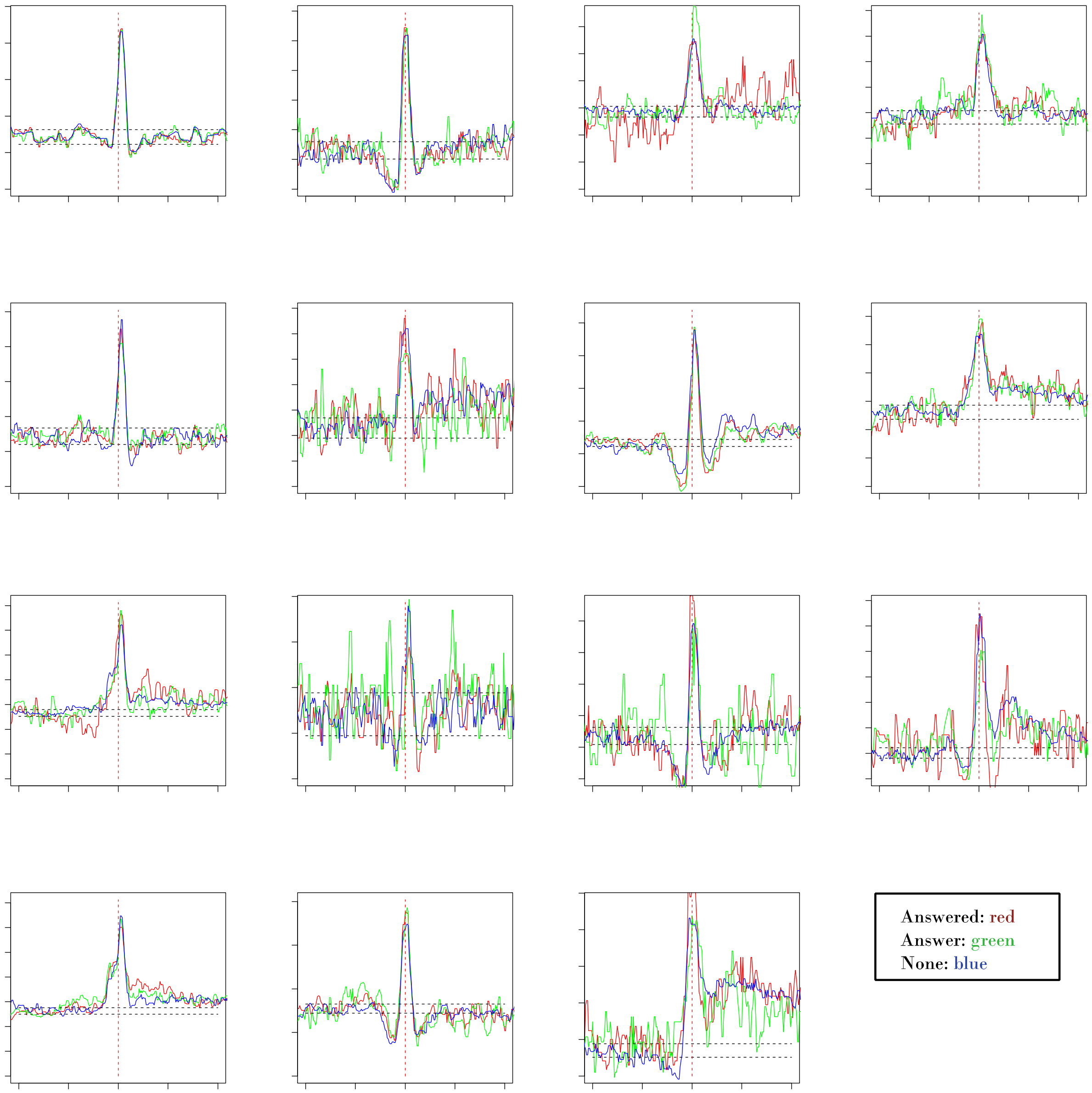

Fig. S5. Neuronal activity during stack or tet calls used in different contexts. Calls (stacks and tets) of all females, that established antiphonal communication with one of the males, are divided in three groups: Female calls “answered” by a male call, female calls used as an “answer” (green lines) and female calls used in “none” (blue lines) of these contexts. Neuronal signals underlying the respective call category are aligned to all three versions of usage in a peri stimulus time histogram. Red lines: neural

association to ‘answered calls’, green lines: neural association to ‘answer calls’ and blue lines: neural association to calls in no recognizable context. The association between neuronal signals and respectively used call category are not different. Female 4 showed all possible combinations (stack-stack, stack-tet, tet-tet and tet-stack), but at least one combination is presented for the other experimental females.
